# Exploring the Impostor Phenomenon Among Korean Daycare Directors: A Grounded Theory Approach

**DOI:** 10.3390/bs15050565

**Published:** 2025-04-22

**Authors:** Ji-Hyeon Choi, Young-Eun Lee

**Affiliations:** Department of Early Childhood Education, College of Social Science, Gachon University, Seongnam 13120, Republic of Korea; jihyun111111@gachon.ac.kr

**Keywords:** Korean daycare director, impostor phenomenon, grounded theory, ecological systems theory, perfectionism

## Abstract

The impostor phenomenon, characterized by self-doubt and an external attribution of success, significantly impacts daycare center directors, influencing their leadership effectiveness and childcare quality. This qualitative study aims to explore how the impostor phenomenon manifests among Korean daycare center directors within an ecological framework. Semi-structured interviews were conducted with 15 Korean daycare center directors using grounded theory methods. Analysis identified the phenomenon across cognitive, emotional, and behavioral dimensions, revealing both negative self-perceptions and strategic, perfectionism-related behaviors consistent with previous research. This study proposes a contextual model based on Bronfenbrenner’s ecological systems theory, illustrating interactions at macrosystem, exosystem, mesosystem, microsystem, and chronosystem levels, with a detailed paradigm model further explaining microsystem and chronosystem interactions. These findings contribute to clarifying and contextualizing the impostor phenomenon, particularly highlighting situational influences and strategic manifestations. This research provides a foundation for future studies in South Korean contexts and practical insights for developing targeted leadership support programs for daycare center directors.

## 1. Introduction

Owing to rapid economic growth, fierce competitiveness and hierarchy have become deeply entrenched in Korean society. In South Korea’s hierarchical society, psychological pressure is further exacerbated when individuals feel that their performance is not sufficiently recognized, with those in leadership positions experiencing the impostor phenomenon more strongly (Y. S. Choi & Choi, 2020; Rohrmann et al., 2016). The impostor phenomenon is a psychological condition in which individuals persistently doubt their abilities and attribute success to external factors (Clance & Imes, 1978). It is associated with increased depression, anxiety, burnout, and job dissatisfaction (McGregor et al., 2008; Vergauwe et al., 2015) and is particularly common among high-achieving women (Clance & Imes, 1978). This may be even more pronounced in female-dominated occupations. According to the Seoul Metropolitan Government (2023), childcare center directors are overwhelmingly women, with 4346 (98.3%) compared to 74 (1.7%) men.

Although previous research on the impostor phenomenon has been conducted extensively in Western contexts including North America and Europe, studies in some Asian countries remain relatively fewer. These Western studies have primarily focused on specific professional populations, such as those in medicine (LaDonna et al., 2018; Chodoff et al., 2023; Chakraverty, 2024b), engineering (Maji, 2021; Chakraverty, 2021, 2022, 2024a), and among doctoral students (Cope-Watson & Betts, 2010; Cisco, 2020). In East Asia, a few studies have begun to explore this phenomenon. For instance, in China, Xu (2020) examined the relationship between implicit intelligence and impostor feelings among adolescents, while Liu et al. (2023) investigated the mediating role of impostor experiences between perfectionism and depression in college students.

In South Korea, however, empirical research on the impostor phenomenon remains scarce. To date, only a small number of quantitative studies have been conducted—for example, G. Y. Kim and Lee (2023) focused on athletes, and Byun (2024) examined female university faculty members, both using the term “impostor syndrome”. Notably, there is no prior research specifically addressing the impostor phenomenon among Korean daycare center directors. Existing qualitative studies on daycare center directors in Korea have instead explored different topics, such as institutional challenges (Cha & Kwon, 2011; E. J. Lee & Park, 2018; Kang et al., 2019; M. K. Yoon & Bae, 2021) and leadership-related concerns (Cho, 2011; Oh, 2012; H. J. Yoon & Heo, 2012).

Feenstra et al. (2020) argued that instead of viewing impostorism as a personality trait, it is crucial to explore ways to reduce impostor emotions by identifying the social and contextual factors that influence it. Building on this perspective, Tewfik et al. (2025) emphasize the importance of clarifying the conceptual core of the impostor phenomenon—particularly the cognitive belief that others have an inflated view of one’s competence—while also distinguishing between enduring trait-like tendencies and situationally triggered impostor experiences. Leonhardt et al. (2017) further contribute to this nuanced understanding by distinguishing between internalized impostor feelings and more performative, strategic expressions of self-doubt, which may be driven by social expectations or impression management goals. These insights underscore the need to explore how impostor feelings emerge in relation to professional context, cultural expectations, and personal disposition.

Therefore, the primary purpose of this study is to explore the impostor phenomenon among Korean daycare center directors and to develop a contextual model grounded in Bronfenbrenner’s ecological systems theory. This model illustrates how the phenomenon unfolds across multiple levels—including the macrosystem, exosystem, mesosystem, microsystem, and chronosystem. In particular, the paradigm model developed in this study explicates dynamics within the microsystem and chronosystem, serving as an analytical component that complements the broader contextual framework.

Directors in Korean daycare centers frequently navigate complex relationships with parents, whose expectations and communication styles may shape the directors’ sense of leadership efficacy. These relational and contextual pressures can interact with internalized self-doubt and contribute to the manifestation of the impostor phenomenon.

To investigate this, the study seeks not only to examine how the impostor phenomenon is experienced by Korean daycare center directors, but also to construct a contextual model that captures the socio-ecological dimensions of these experiences.

The research questions of this study are as follows:

1. How does the impostor phenomenon manifest among Korean daycare center directors?
2. How can the contextual model of the impostor phenomenon among Korean daycare center directors be described?

## 2. Materials and Methods

### 2.1. Research Approach

This study employed a combination of Strauss and Corbin’s (1998) systematic grounded theory and Bowers and Schatzman’s (2016) dimensional analysis to examine the impostor phenomenon among Korean daycare center directors. Systematic grounded theory, a specific approach within grounded theory methodology, provides a structured framework for exploring and explaining the subjective meanings individuals assign to their experiences and the processes through which these meanings emerge (Creswell, 2017). Dimensional analysis, on the other hand, is an independent methodological approach rooted in grounded theory that focuses on identifying the multidimensional nature of a phenomenon and examining the contextual relationships among its core dimensions (Carlin & Kim, 2019). By integrating these two methodologies, this study analyzed the subjective meanings experienced by directors and explored how the impostor phenomenon manifests and evolves in diverse social and psychological contexts. This combined approach enabled a deeper explanation of the emergent processes and the complex structure underlying the impostor phenomenon, providing a clearer understanding of its meaning in relation to the directors’ lived experiences.

In addition, this study adopted a spiral model of analysis, which allowed categories and theoretical frameworks to evolve through continuous engagement with the data. As the data collection and coding progressed, preliminary categories were revisited and refined in response to new insights, allowing theory and interpretation to co-evolve throughout the research process (Cohen et al., 2017). This recursive engagement between data and emerging theory enhanced both the depth and rigor of the analysis.

### 2.2. Participants

This study included 15 Korean daycare center directors, all of whom were female (Table 1). Participants ranged in age from 41 to 61 years, with between 5 and 34 years of experience as directors. Given the study’s focus on internalized professional experiences, it was essential to ensure that participants clearly understood the central phenomenon under investigation.

The term “impostor phenomenon” was introduced as the main topic of the study, and participants were provided with sufficient explanations through video materials and verbal briefings prior to the interviews to ensure a clear and consistent understanding of the concept. The video content included a definition of the impostor phenomenon, along with contextualized examples relevant to Korean society. Only individuals who acknowledged having experienced the impostor phenomenon in the course of their professional roles participated in the interviews. This selection criterion ensured that the data collected accurately reflected participants’ lived experiences and contributed to a deeper understanding of how the impostor phenomenon unfolds within specific social and cultural contexts.

### 2.3. Ethical Issues

This study was conducted with full consideration of participants’ ethical rights and was approved by redacted. Prior to the interviews, the purpose and procedures of the study were explained to the participants, including assurances of confidentiality regarding personal information and interview contents. Participation was voluntary, and written informed consent was obtained from all participants before interviews. Participants were informed that they could withdraw from the study at any time without penalty. All interviews were audio-recorded with participants’ explicit consent, and key transcripts were reviewed for analysis.

### 2.4. Procedure and Analysis

We collected data through face-to-face interviews with the participants, each lasting approximately 60 min. One or two follow-up interviews were conducted at a later date, supplemented by additional non-face-to-face interviews conducted via phone or online.

The interviews were transcribed using CLOVA Note, an automated speech-to-text tool, resulting in approximately 156 pages of transcriptions. These data were analyzed through iterative coding and review. Using Strauss and Corbin’s (1998) systematic grounded theory approach, an inductive qualitative research method, we developed an exploratory model based on empirical data. The analysis followed a three-step coding procedure—open coding, axial coding, and selective coding—to identify major categories and subcategories that explain how the impostor phenomenon manifests among Korean daycare center directors.

In the first step, open coding was conducted, involving line-by-line analysis to generate concepts closely aligned with the data. During this process, memos were drafted to organize and structure the analysis, leading to the identification of axes for further exploration. In the second step, axial coding, patterns of actions were categorized by linking the conditions and consequences of the concepts through constant comparison. Theoretical memos further refined these axes, resulting in the emergence of a core category. In the final step, selective coding ensured consistency and coherence in the analysis. This structured, multi-phase approach facilitated a comprehensive interpretation of participants’ experiences and strengthened the analytic rigor of the study (Morse, 2000).

In parallel, the study employed dimensional analysis to explore the phenomenon’s multidimensional nature within the ecological systems framework. Dimensional analysis, an independent methodological approach rooted in grounded theory, focuses on how phenomena are contextually constructed through various dimensions. This lens allowed us to examine how the impostor phenomenon develops through interactions across ecological layers, from institutional to interpersonal. The resulting contextual model illustrates how ecological factors contribute to the emergence and trajectory of impostor emotions in daycare center leadership.

Finally, the study adopted a spiral model of analysis to synthesize findings from both the literature review and interview data. An initial theoretical framework was established based on prior literature, which was subsequently refined through data-driven analysis. This iterative process of revisiting and adjusting theory ensured an increasing theoretical alignment and depth over time (Bryman, 2016). The dynamic interaction between the literature, data, and emerging interpretations enhanced the overall completeness and credibility of the findings.

## 3. Results

To investigate the manifestation of the impostor phenomenon as experienced by Korean daycare center directors, the collected data were continually compared and analyzed to group similar concepts. The categorization results are presented in Table 2.

To better reflect the multifaceted experiences described by participants, the findings are presented across cognitive, emotional, and behavioral dimensions. This structure facilitates clarity while capturing both self-doubting and strategically adaptive responses.

### 3.1. Open Coding

**Mental health**

Korean daycare center directors reported feeling discouraged when their efforts were unrecognized by parents and experiencing internal conflict when they questioned their suitability for their role as directors. Similarly, individuals experiencing the impostor phenomenon often struggle with depression, anxiety, and lethargy when they feel their efforts fall short of expectations or when they question their qualifications (McGregor et al., 2008).

***Despondency***

*“Sometimes I get angry and upset when I have worked hard and been sensitive to safety issues in childcare all day and the parent does not acknowledge it, especially when I have to admit I did something wrong where I did not do anything wrong”. *

*(D3)*

***Internal conflict about the job***

*“It makes you think, “Oh, this is not working, even though I am working so hard”, and now sometimes you have one too many negative experiences with parents that make you rethink this business”.*

*(D1)*

**Personality**

Directors with perfectionist personalities showed varied work performance and stress levels, which was linked to the impostor phenomenon. This connection aligns with findings by D. Yoon (2022), who stated that perfectionism can be categorized into four types: avoidant, supervisory, self-blaming, and stable. In this study, the directors exhibited supervisory, self-blaming, and stable forms of perfectionism. Supervisory perfectionists impose their high standards on others, which can cause stress to those around them, whereas self-blaming perfectionists value other people’s standards more than their own and tend to focus on outcomes rather than the process. In contrast, stable perfectionists selectively pursue perfection in essential tasks, utilizing the positive aspects of perfectionism without fearing mistakes or failures.

***Perfectionist personality***

Supervisory perfectionists excessively interfered with teachers’ work to bring it up to their own standards, which resulted in the common anxiety and self-doubt associated with the impostor phenomenon, as they viewed the failure to meet these high standards as evidence of a lack of competence.

*“I am not just perfect for myself, I am perfect for others, and I think that is why the teachers here are more meticulous and detailed and good at it. I did not know it, but I think I am the kind of person who imposes what is in me on others”.*

*(D10)*

Self-blaming perfectionists struggled to accept their imperfections, became overly sensitive to parents’ judgment, and excessively criticized themselves. This was closely related to the impostor phenomenon, as they frequently doubted their qualities and judged themselves harshly.

*“I am a perfectionist, and I go back over my mistakes to make sure they do not cause any damage, so I do not sleep well at night during events”.*

*(D8)*

In contrast, stable perfectionist principals are able to reduce both their own stress and that of the teachers by pursuing perfection only in important tasks, thus alleviating anxiety related to the impostor phenomenon.

*“When I do the paperwork, I try to do it perfectly, but I think I try not to do it too perfectly because if I do it perfectly, how hard is it going to be for everyone else?”*

*(D6)*

**Personal beliefs**

Finally, directors set expectations for their roles based on their beliefs about needing to prove their expertise as education experts or as executives for managing budgets efficiently in running a daycare center. Psychological pressure tends to intensify when their performance falls short of these expectations.

***Beliefs as an education expert***

*“I would like to see an objective evaluation of the word expert, not in terms of years, but in terms of actual skill. I have been an expert for decades. But if I cannot show that when I ask myself, ‘What do you know how to do?’ then I think I am not an expert”.*

*(D2)*

***Beliefs as an executive***

*“I consider myself more of an operator than an educator, as I have been a director for much longer. I see myself as an entrepreneurial director, essentially a businessman, responsible for recruiting children and managing finances. For instance, if the income is 100, I need to allocate percentages from that amount, assess the value provided to the children, and evaluate the effectiveness of that value. It is not just about how much money I make but also about ensuring the value I gain aligns with the work I put in”. *

*(D7)*

**Sociocultural characteristics of South Korea**

Korean daycare center directors face significant pressure from parents’ expectations to manage daycare centers in this highly competitive environment. This heightened pressure stems from South Korea’s ultra-low birthrate and reinforces the tendency for parents to invest heavily in a single child to ensure their success in an increasingly competitive society (J. W. Lee et al., 2018).

***Daycare center management challenges in Korean society***

In this study, directors raised in a traditional Confucian society with collectivistic tendencies experienced conflicts arising from the individualistic tendencies of the parents, who are also their children’s peers. According to U. Kim (2006), South Korea was historically a collectivist society that prioritized the stability and interests of groups, such as the family and state, over the individual until the end of the 19th century. This was largely due to the influence of Confucian culture, which valued group harmony above individual interests. However, with the introduction of Western democracy and industrialization in the early 20th century, individualism, emphasizing individual autonomy and rights independent of group needs, began to spread (U. Kim, 2006). S. W. Yoon (2016) also noted that South Korea’s rapid economic growth has created a society where several ideologies coexist, including meritocracy, which fosters competition, and authoritarianism, which emphasizes hierarchy.

*“It is stressful because we are constantly competing and we are constantly having to surface the things that we are doing well, and it is a social atmosphere”.*

*(D13)*

*“I do not think there should be individualism when looking at children, but nowadays, parents are too individualistic, so it is difficult for institutions to live in a group”.*

*(D6)*

Additionally, this study found that growing up in this traditional Confucian society led to the development of introverted and principled personalities. Shin and Jeong (2002) further highlighted that traditional Korean society is rooted in an authoritarian culture based on Confucian values, which has reinforced patriarchy and hierarchy. These Confucian traditions have entrenched power structures within society, with absolute respect for paternal authority in the family and hierarchical relationships between teachers and students in education.

*“My dad was a teacher, so I grew up getting beaten up a lot, and it really affected my character and my self-esteem, so I do not like to be in front of anybody, and I am very shy”.*

*(D4)*

**Policy**

***Frequent curriculum changes***

Daycare center directors continued to face challenges in implementing a play-based curriculum, particularly due to parental misunderstandings about the nature of learning through play. The “Nuri Curriculum for Ages Three to Five”, implemented in March 2013, was criticized for limiting toddlers’ opportunities to play by adopting teacher-centered teaching methods (Lim, 2017). In response, the play-centered curriculum, introduced in March 2020, focuses on toddler-led learning through play, emphasizing child agency in the learning process (Ministry of Education & Ministry of Health and Welfare, 2019). However, according to E. Y. Kim et al. (2020), while parents recognized the value of a play-centered curriculum, they expressed concerns that their children might simply play at daycare centers and face difficulties adapting to elementary school.

*“It is hard to put play-centeredness into practice, it is very hard, but you have to do it, so you have to tell the teachers, you know, this is wrong, this is how it should be done, this is how it should be done, but then when you are in the field, it does not go the way it should go in theory, so I think that is the hardest part”.*

*(D4)*

*“I think the process of applying play-centeredness makes you think a lot about whether it looks like you are just playing to the parents, or whether it looks like you are not interested in the child”.*

*(D1)*

**Impostor phenomenon cognitive aspects**

In this study, directors blamed themselves for poor counseling and conflict resolution skills in their relationships with parents, underestimated their abilities as directors, and attributed their success to luck or external factors. This tendency aligns with findings on the impostor phenomenon, where individuals often attribute their achievements to external factors or luck rather than taking credit for them (McGregor et al., 2008).

***Self-devaluation***

*“I think if I would have looked at it a little bit more, if I would have coached the teachers a little bit more when the parents were point, and I think there is also a sense of self-blame now that we are problem solving, when signing the teachers like this, we would not have gotten to this end there’s a complaint like that, when we are solving this, it would be nice if it worked out, but when it does not, it is because I am not good enough”. *

*(D14)*

***Luck***

“When I hear positive things about me from parents, I actually look at myself and think to myself that maybe they are giving me too much credit because I did not do enough to deserve it”.

(D7)

**Impostor phenomenon emotional aspects**

In this study, directors experienced emotional withdrawal due to perceived inadequacies in handling situations with parents, which led to low self-esteem. Furthermore, they faced emotional exhaustion stemming from conflicts in their relationships with parents and exhaustion from taking on extroverted roles that conflicted with their introverted personalities. Research has shown a strong link between the impostor phenomenon and low self-esteem (Chrisman et al., 1995), as well as its association with excessive stress and emotional burnout (Hutchins & Rainbolt, 2017).

***Low self-esteem***

*“I feel like I lose my self-esteem when I feel like I am not good enough, and I feel like I am not good enough to cope like this”.*

*(D10)*

***Emotional exhaustion***

*“I am usually an introvert, but when I am at the daycare center, I become more socially active. During events or when parents come to visit, there are tasks that I need to take the lead on. After handling those, I feel emotionally drained when I get home”.*

*(D15)*

**Impostor phenomenon behavioral aspects**

In this study, directors worked tirelessly to mask their perceived lack of competence during parent counseling sessions, which led to excessive complaint handling and over-preparation for meetings with parents. They also maintained an outwardly confident demeanor while concealing their inner insecurities. This aligns with Ferrari and Thompson’s (2006) findings that individuals with impostor syndrome often hide their anxiety behind a façade of confidence.

***Overwork***

*“Especially with parents, I am worried about revealing my ignorance to them, so I end up staying up all night looking for resources, studying, and dealing with parents before the consultation. When dealing with parents, I feel like I have to keep proving and showing them that we are different. I double- and triple-check what the teachers have done to make sure I do not miss any details”.*

*(D1)*

***Fake***

*“I think I have a little bit of a timid side to me, and sometimes I get intimidated if I am not ready or if someone’s doing a better job than I am, and you cannot do that with parents, and I am supposed to be the one talking and leading, so I think I am just trying to make myself look good in my head”. *

*(D9)*

**Individual effort**

As they experienced the impostor phenomenon in their relationships with parents, the directors also sought self-improvement, such as by attending graduate school to increase their theoretical expertise or undergoing training to complement their skills. They also engaged in leisure activities such as traveling, exercising, and reading to relieve stress from their work, and found emotional comfort in religious activities to restore their psychological well-being. These efforts reflect directors’ attempts to overcome feelings of inadequacy and regain a sense of competence.

***Self-development***

*“I started my master’s because I was afraid of falling behind others and because of what my parents thought of me. I am a business major, so I always feel like I am lacking in my education”.*

*(D1)*

*“I am learning Instagram, I am learning Excel, I am learning PPT, I am learning how to make videos”. *

*(D8)*

***Leisure and hobbies***

*“I de-stress by going to the mountains or playing golf, or I will watch a really good show for 10 h without thinking about anything, because there is nothing I can do about it, and it is only going to get better with time”. *

*(D11)*

***Religious life***

*“I think that is what gives me the strength to get through the hard stuff, because I am a Christian, I pray, I go to church, I cry, and then I pick myself up and get back on my feet”.*

*(D10)*

**Social support**

Emotional support from close people was also an important intervening condition.

***Peer director***

In terms of relationships with peer directors, some directors who passively participated in activities felt intimidated when their abilities were compared with those of their peers. However, directors who had close relationships and actively communicated with their peers received emotional support and professional help to alleviate their job stress.

*“The directors have similar experiences with parents, so we talk to each other when we are upset and get advice when we are struggling, and I think it is great to get wisdom”.*

*(D4)*

***Family***

In terms of family relationships, some principals gained emotional support by communicating with their spouses and children, which helped mitigate the impostor effect.

*“I talk to him a lot, and I am just like, today was like this, today was like that, and now I am just talking about how hard it was for me because of him, and he is just kind of talking from my side, from my teacher’s side, from our daycare center’s side, and he is kind of like, it is not your fault, it is just that he’s weird, and then I am running it, and I am like, this is not working, this is hard, and then he’s like, he will do this, and then he will do that, and then I am kind of relying on him a lot”. *

*(D5)*

**Differences in demand stability for daycare centers**

The stability of demand at daycare centers also played a crucial role. Directors with stable enrollment, either due to new apartment buildings nearby or the institution’s management strategy, felt comfortable in their relationships with parents and experienced less of the impostor phenomenon. In contrast, the impostor phenomenon was intensified in centers experiencing difficulty in recruiting children because of competition.

***High-demand organizations***

*“Because it is a new complex, we have so many people coming in from outside, I do not think we’ll have a problem recruiting for the next few years”.*

*(D7)*

***Low-demand organizations***

*“It is the second half of the year, so we have to prepare for kindergarten recruitment, and the situation we are in right now is that there is a big kindergarten with 300 students around us, so there is a lot of things that we cannot do because we are a small childcare center. I think there is anxiety and discomfort because of those things”. *

*(D13)*

**Regional differences**

***The difference between old and new cities***

Finally, regional differences also contributed to the impostor phenomenon. Many more experienced directors had moved more than once; as a result, they became aware of the differences in educational expectations and needs between older and newer urban parents. In particular, they had difficulty adapting to parents in newer cities with higher educational expectations and demands. However, over time, they gradually adapted as they understood the needs of parents and found appropriate strategies, thus gradually easing the impostor phenomenon.

*“Where I was before, it was actually not a big deal as long as there was trust, but when I came to this new apartment complex, there is a lot of superiority here. There is a lot of, ‘I want you to do this for me because I want my child to be this big.’”*

*(D7)*

*“I think when you get a new apartment and you move in, it takes a little bit of time to adjust. I think the way parents treat teachers or daycare centers, I think that level gets less and less over the years, and you get better and better at accepting a lot of demands”.*

*(D15)*

**Cognitive changes**

Directors who experienced the impostor phenomenon underwent a cognitive shift characterized by pursuing a renewed sense of intrinsic motivation and emphasizing internal happiness and self-worth over material success. This shift helped them overcome the imposter syndrome by relieving the pressure they felt in their relationships with parents and shifting their thinking to recognizing their own efforts and value.

***Pursuing intrinsic motivation***

*“Success used to be my most important goal, but nowadays, I have come to value my happiness more than success, which has freed me up to enjoy the here and now”.*

*(D11)*

***Change in value***

*“You’re like, ‘I gave you my best and you cannot accept it, so there is nothing more I can do for you, and that is your fault, and I am not ashamed of that at all,’ and if you perceive me that way even though I gave you my best, I am like, ‘Well, I’m just going to keep working hard and maybe someone else will.’ It is changed a lot”.*

*(D4)*

**Emotional changes**

***The courage of self-acknowledgment***

The directors also experienced an emotional transformation, having the courage to acknowledge their imperfections and accept themselves as they were. This helped them find emotional stability in the process of overcoming the impostor phenomenon, reducing their anxiety and fear regarding their role as directors in their relationships with parents.

*“But now I am just like, there might be some things that I am not good at and I just accept that and I just do the best that I can and I am just like, I am not going to be the best at anything right now, but I am going to gain experience and I am going to be happy with that, and I think that is a lot easier for me”. *

*(D4)*

**Behavioral changes**

***Pursuing work–life balance***

Lastly, directors exhibited a behavioral shift toward work–life balance. Previously, they had worked excessively to meet parental expectations but now strive to balance their personal and professional lives. This shift helped directors overcome the impostor phenomenon and fulfill their roles as directors in a healthier way.

*“I try to take breaks when I am having a hard time so that I can work long hours, and I try to be really good at de-escalating my work, and I think home is just as important as work, so I try not to bring that stress home until the weekend”.*

*(D6)*

**Impostor phenomenon persists**

***Maintaining impostor phenomenon***

Some directors who experienced the impostor phenomenon still felt overwhelmed by increasing responsibilities as they progressed in their careers and continued to put on a confident outward appearance. They remained constantly aware of negative parental evaluations, which kept them on the edge.

*“The more experience I have, the more responsibility I have, and I think the more positions and situations I’m in, the more I try to hide myself and put on a good face”.*

*(D10)*

*“I do not think it is gotten any less nerve-wracking. It is not that it’s easy when you are dealing with parents endlessly, but it is still the same nervousness of what is this person going to think of me, what is this person going to think of our circle, what is this person going to say when they go out and talk to people”.*

*(D14)*

***Reduced impostor phenomenon***

In contrast, some directors found psychological relief from being judged by others, and the impostor phenomenon was mitigated by recognizing their own efforts and value.

*“I feel like I have a strong tendency to try to impress others, but these days I am trying to just be myself and express myself, and I think that is much more authentic and natural”.*

*(D12)*

According to the interview data, Korean daycare center directors experience a range of psychological and contextual challenges, often manifesting as feelings of inadequacy, internal conflict, and stress. These experiences are closely connected to the impostor phenomenon, which appears across cognitive, emotional, and behavioral dimensions. Contributing factors include perfectionist tendencies, internalized beliefs about their professional roles, sociocultural expectations rooted in Korean society, and frequent changes in the educational curriculum. While some of these experiences may reflect deeply internalized impostor feelings, others—particularly perfectionism-driven overwork or role enactment—may be better understood as strategic adaptations to professional norms and parental expectations.

Despite these challenges, some directors strive for self-development and seek emotional support from fellow directors or family members to maintain psychological stability. In addition, variations in regional and institutional contexts lead to differences in how the impostor phenomenon is experienced. Notably, while some directors undergo positive cognitive, emotional, and behavioral changes that help them overcome the impostor phenomenon, others continue to be affected by these feelings over time. To more clearly explain the relationships between these categories and to gain a deeper understanding of how the impostor phenomenon is experienced and navigated, the following section presents an analysis based on the paradigm model proposed by Strauss and Corbin (1998).

### 3.2. Paradigm Model

**Figure 1:**
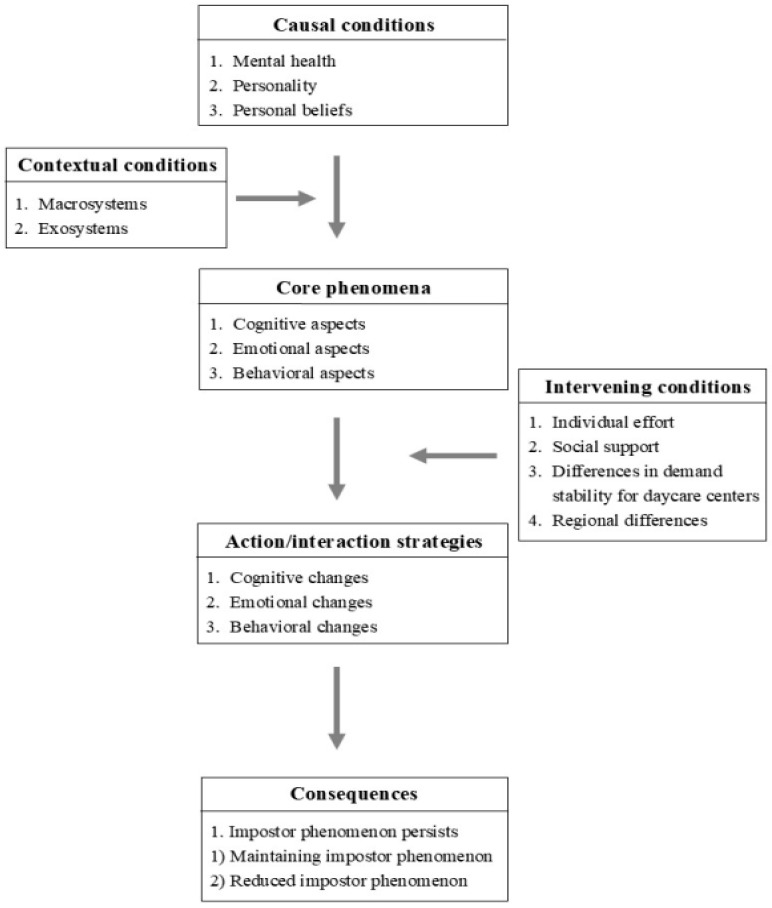
A paradigm model of the impostor phenomenon among Korean daycare center directors.

Strauss and Corbin (1998) proposed a paradigm model centered on a core phenomenon and encompassing causal conditions, contextual conditions, intervening conditions, action/interaction strategies, and consequences. Based on this, connections between the categories were established, as shown in Figure 1. In this study, the paradigm model particularly informs an analysis of microsystem-level stressors and time-based developmental changes—functioning as a dynamic component of the broader contextual model.

### 3.3. Contextual Model

**Figure 2:**
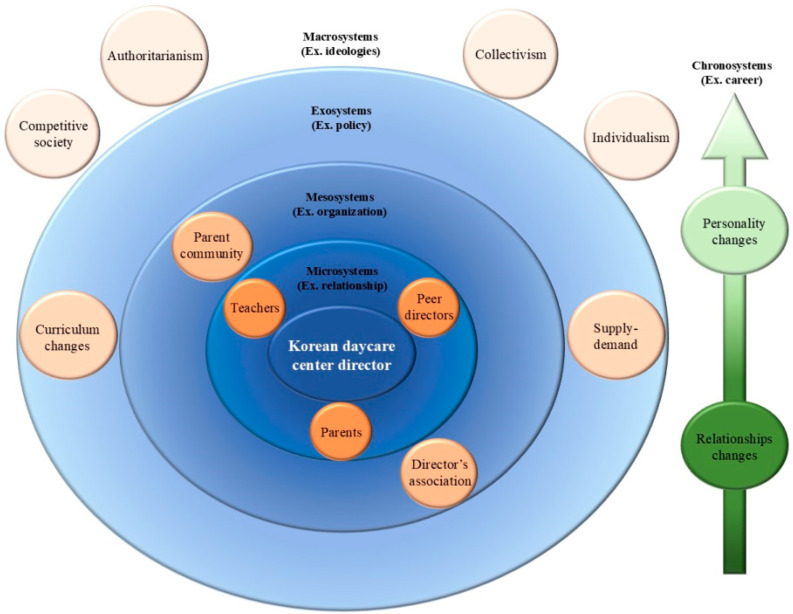
A contextual model of the impostor phenomenon among Korean daycare center directors.

In the preceding section, the impostor phenomenon experienced by daycare center directors was analyzed using the paradigm model proposed by Strauss and Corbin (1998). To better reflect the complex and multilayered nature of the phenomenon, this study emphasizes the use of a contextual model. This model serves as the primary framework for analyzing how the impostor phenomenon manifests across various ecological levels (Figure 2) derived through dimensional analysis, as proposed by Bowers and Schatzman (2016). This approach enabled a systematic exploration of the multiple domains influencing directors’ experiences and interpretations of the impostor phenomenon.

The contextual model of the impostor phenomenon experienced by Korean daycare center directors (Figure 2) is based on Bronfenbrenner’s ecological systems theory (1994). The model includes “macrosystems”, “exosystems”, “mesosystems”, “microsystems”, and “chronosystems”, each of which acts as a factor that compounds and reinforces the impostor phenomenon experienced by the directors.

Among these, the microsystem and chronosystem emerged as especially salient in participants’ narratives. The microsystem captures direct interpersonal dynamics—particularly interactions with parents and peers—where internalized self-doubt and strategic behaviors surface. This is consistent with earlier research highlighting how impostor experiences are shaped through immediate social relationships (e.g., Tewfik et al., 2025; Leonhardt et al., 2017). In contrast, the chronosystem represents the developmental trajectory of impostorism, revealing how impostor feelings may intensify or transform over time. These temporal aspects were more directly illuminated through the paradigm model, which captured participants’ reflections on evolving coping strategies and shifting self-perceptions as they gained experience, transitioned roles, and took on greater responsibilities.

The macrosystem indirectly affected the impostor phenomenon by reflecting the sociocultural characteristics of Korean society. South Korea’s sociocultural context is shaped by a blend of Confucian collectivism, rising individualism, and hierarchical values, alongside a strong emphasis on academic and professional success (U. Kim, 2006; S. W. Yoon, 2016). In addition, the country’s ultra-low birthrate has intensified parental investment in their children, increasing expectations on early childhood education settings (J. W. Lee et al., 2018). The macrosystem in this model represents a competitive society in which authoritarianism, collectivism, and individualism stemming from Confucian culture coexist and interact. This sociocultural context reinforces the impostor phenomenon, as directors experience an increased conflict and stress in their relationship with parents, feeling unworthy. These dynamics are further complicated by Korea’s strong face-saving norms, which discourage public displays of uncertainty and reinforce pressure to appear competent (N. Y. Choi & Miller, 2014).

The exosystem was identified as the play-centered curriculum, a policy-driven curriculum for Korean early childhood education. The Nuri Curriculum, implemented in 2013, faced criticism for its teacher-centered approach that restricted children’s play opportunities (Lim, 2017). In response, a revised play-centered curriculum was introduced in 2020, emphasizing child-led learning and promoting children’s agency in the educational process (Ministry of Education & Ministry of Health and Welfare, 2019). In this study, the directors found it difficult to apply the new curriculum; in particular, they were concerned that parents would misunderstand the meaning of learning through play and see it as mere play. In addition, differences in demand stability due to regional characteristics and institutional management strategies emerged. Directors with a stable enrollment felt comfortable in their relationships with parents, whereas those struggling to recruit children felt pressured.

The mesosystem was represented by the parent communication community (mom café) and the director’s association. In this study, we found that directors who frequently checked mom cafés, a parent communication community, were more concerned and anxious about negative posts or comments from parents, whereas those who did not check them were less affected. Mom cafés are online communities where parents share information and opinions about raising children, often using them to discuss their experiences with daycare centers. According to S. B. Park (2019), posts on mom cafés may contain untrue or exaggerated information, which can quickly spread and damage the reputation of daycare centers. In particular, when parents publicly post complaints or grievances on mom cafés, it can have a serious impact on child enrollment and the recruitment of new children. As a result, directors tend to be cautious and take measures to prevent such situations. Some directors who were not actively involved in the daycare center association felt intimidated by comparisons with their peers. Conversely, directors who had close relationships with their peers and actively communicated with them received emotional support and professional help to alleviate job stress.

The microsystem was represented by relationships with peer directors, teachers, and parents, with whom the director directly interacts. Some directors maintained positive relationships with their peers, while others interacted with them in a more formal or business-like manner. In their relationship with teachers, the director found it difficult to exert authority due to teacher turnover and problematic behaviors. In addition, collectivistic directors tried to build positive relationships with teachers but were frustrated by their individualistic tendencies. In terms of relationships with parents, directors aimed to maintain positive relationships but sometimes treated parents more like customers whose needs needed to be met rather than as collaborators in the care of infants and toddlers.

The chronosystem is represented by changes in the directors’ personalities and relationships over time. The extroverted personalities gradually became more introverted as their responsibilities as directors increased and their relationships were reduced to minimize stress. As the directors’ ability to filter information improved, their relationships became narrower, focusing more on those who could provide quality information than on a broad range of people. These evolving strategies may also reflect a shift from externally oriented impression management toward more intrinsic self-validation—signaling a continuum between strategic impostor responses and internal transformation over time.

## 4. Discussion

Tewfik et al. (2025) and Leonhardt et al. (2017) emphasize that impostor-like behaviors are not always rooted in internalized self-doubt but may also reflect situationally adaptive strategies or impression management. Drawing on these insights, we interpret some of the participants’ behaviors—such as over-preparation or role-playing confidence—not merely as symptoms of impostorism, but as responses shaped by contextual expectations. This interpretive lens is further supported by the ecological and dimensional frameworks employed in this study.

The purpose of this study was to explore the impostor phenomenon among Korean daycare center directors and to present a contextual model that explains how this phenomenon is shaped within multilayered environmental systems.

The results from this study reflect the multifaceted nature of the impostor phenomenon, aligning with prior research that identifies both its negative and adaptive aspects (Tewfik et al., 2025). Jackson (2018) defined the leadership impostor phenomenon as the inability of successful leaders to internalize their achievements, attributing them to luck or external factors while harboring a fear of failure. The cognitive aspects of the impostor phenomenon, where directors devalued themselves and credited success to external factors, support Jackson’s conceptualization. Additionally, this study supports Jackson’s findings on perfectionism by showing that directors with perfectionist tendencies often experienced a heightened psychological pressure.

Feenstra et al. (2020) found that female leaders often experienced emotional burnout and maintained a confident outward demeanor to mask their inner feelings of power insecurity. Similarly, our findings indicate that Korean daycare center directors experienced emotional exhaustion and low self-esteem while simultaneously engaging in masking behaviors—an alignment with previously observed behavioral aspects of the impostor phenomenon. At the same time, consistent with Leonhardt et al.’s (2017) distinction between internalized impostor feelings and strategic self-presentation, some directors’ behaviors—such as over-preparation and image management—can be interpreted not only as symptoms of impostorism but also as deliberate strategies influenced by professional expectations and perfectionist tendencies. These interpretations suggest that the impostor phenomenon may have adaptive dimensions, particularly in the context of leadership, where perfectionism may serve both as a psychological burden and a performance enhancer depending on individual traits and situational demands.

Extending prior work that has focused largely on Western and some Asian contexts, our study confirms the cultural adaptability of the impostor phenomenon by situating it within South Korea’s unique sociocultural context. This underscores the global relevance of impostorism and highlights the impact of national-level sociocultural forces—such as Confucian hierarchical values, educational competitiveness, and parental expectations—on the phenomenon. These structural and cultural elements, reflected in the macrosystem layer of our contextual model, help explain why the impostor phenomenon in Korea may carry more negative emotional weight and stress compared to other contexts. In particular, social norms around face-saving, gendered leadership expectations, and pressures from a low birthrate society reinforce internalized doubts and performance anxiety. Gendered leadership expectations may pressure female directors to uphold idealized standards of authority, while the low birthrate context intensifies parental demands on early childhood institutions. Among these, face-saving norms in particular often function as social imperatives that limit emotional expression and increase the psychological cost of perceived failure (N. Y. Choi & Miller, 2014).

A notable contribution of this study is its tripartite framework, which distinguishes cognitive, emotional, and behavioral aspects of the impostor experience. While earlier studies have often presented fragmented sub-factors such as “luck”, “fake”, and “discount”, this study integrates those under broader psychological dimensions to better clarify the phenomenon, as called for by Tewfik et al. (2025).

Furthermore, this study shows that the impostor phenomenon may evolve over the course of a director’s career. Whereas early-career directors may exhibit overt behavioral masking due to inexperience, later-career directors in this study displayed more internalized cognitive conflicts while maintaining a professional façade. This developmental trajectory aligns with the chronosystem emphasis in our contextual model and suggests that tailored interventions should consider career stage.

Crucially, the ecological model developed in this study contributes to the expanding call for more context-sensitive approaches to impostorism (Feenstra et al., 2020). By applying Bronfenbrenner’s (1994) ecological systems theory, the study situates impostorism within multiple interactive systems—ranging from societal norms (macrosystem) to immediate professional relationships (microsystem) and longitudinal development (chronosystem). The microsystem, in particular, encapsulates peer and parent dynamics that closely align with prior research (Tewfik et al., 2025; Leonhardt et al., 2017), while the chronosystem dimension emphasizes the evolving nature of impostor responses—first surfaced through the paradigm model and elaborated through dimensional analysis.

In this way, the contextual model complements the paradigm model by revealing the ecological layers that frame impostorism and by offering a dynamic perspective on how directors navigate internal doubt and external expectations over time. This approach allows for deeper insights into the psychosocial mechanisms underlying impostorism in leadership roles.

Taken together, these findings offer valuable directions for future research and practice. The nuanced understanding of impostor phenomenon developed here could inform the design of counseling programs, leadership training, and institutional policies to support directors’ psychological well-being and professional development.

## 5. Conclusions

This study employed a grounded theory approach to explore the impostor phenomenon among Korean daycare center directors, offering an in-depth and context-sensitive understanding of how self-doubt, emotional strain, and strategic behavior unfold within complex social environments. Drawing on ecological systems theory and dimensional analysis, the study presented a contextual model that highlights how the impostor phenomenon is shaped by intersecting psychological, relational, and cultural forces.

By identifying cognitive, emotional, and behavioral dimensions of impostorism and mapping them across macro-, exo-, meso-, micro-, and chronosystem layers, this study contributes to a more nuanced conceptualization of the impostor phenomenon. In particular, the chronosystem dimension captured the evolving nature of impostor experiences over time, while the microsystem clarified how everyday interpersonal dynamics reinforce internalized self-doubt or promote resilience.

This research broadens the scope of qualitative inquiry into impostorism and deepens our understanding of its manifestations in South Korean leadership contexts. The findings may inform the development of tailored support systems—such as counseling programs and leadership training—designed to address both the psychological and sociocultural dimensions of impostor experiences in educational and care-related professions.

## Figures and Tables

**Table 1:** Participant characteristics.

Code	Gender	Age (Years)	Director Experience (Years)
1	Female	41	5
2	Female	50	12
3	Female	51	17
4	Female	47	19
5	Female	44	10
6	Female	51	14
7	Female	60	22
8	Female	61	34
9	Female	58	22
10	Female	60	27
11	Female	54	19
12	Female	51	12
13	Female	53	22
14	Female	45	15
15	Female	47	11

**Table 2:** Concepts, subcategories, and categories derived from the interviews.

Category	Subcategories	Concepts
Mental health	Despondency	The emotions felt when one’s efforts are denied
	Internal conflict about the job	Internal conflict arising from the diminishing professional status of directors
		Internal conflict due to a director’s job insecurity
Personality	Perfectionist personality	Excessive interference with center teachers’ work to bring their performance up to their own standards
		Unable to accept their own imperfections and putting too much pressure on themselves
		A personality that selectively strives for perfection in the things that matter to them
Personal beliefs	Beliefs as an education expert	Belief that you need to prove your expertise as an education expert
	Beliefs as an executive	Operational belief in using the budget efficiently
Sociocultural characteristics of South Korea	Daycare center management challenges in Korean society	Challenges of operating daycare centers due to the supply–demand imbalance caused by the ultra-low birthrate and competitive society
		A society where the patriarchal values of traditional Confucian culture, vertical hierarchy, and individualism coexist
		Challenges of meeting the diverse needs arising from the generation gap and individualistic parents in a rapidly changing Korean society
Policy	Frequent curriculum changes	Difficulty adapting a frequently changing curriculum to the field
		Concerns that the recent changes to the curriculum (play-centered curriculum) will be perceived by parents as play rather than education
Impostor phenomenon cognitive aspects	Self-devaluation	Self-blame and an underestimation of parent counseling skills
		Self-blame and an underestimation of infant and toddler problem behavior coping and conflict resolution skills
	Luck	Attributing your success to luck or external factors
Impostor phenomenon emotional aspects	Low self-esteem	Low self-esteem due to negative experiences with parents
		Low self-esteem due to a perceived lack of competence
	Emotional exhaustion	Emotional exhaustion from conflict with parents
		Emotional exhaustion when an introverted director takes on an extroverted role
Impostor phenomenon behavioral aspects	Overwork	Overworking to fill skill gaps
	Fake	Acting confident to hide your insecurities
Individual effort	Self-development	Attending graduate school for theoretical expertise
		Participation in director training to supplement skills needed in the field
	Leisure and hobbies	Relieve stress by traveling, exercising, and reading
	Religious life	Using faith for emotional comfort and overcoming challenges
Social support	Peer director	Whether you are active in an association
		Whether you receive emotional support from a peer director with whom you have a personal connection
		Whether a personal connection with a peer director helps you in your work
	Family	Whether emotional support is received through communication with a spouse and children
Differences in demand stability for daycare centers	High-demand organizations	Stability in enrollment due to geography or institutional business strategy
	Low-demand organizations	Difficulty recruiting kindergarteners due to competition from neighboring institutions
Regional differences	The difference between old and new cities	Older city parents’ trusting and collaborative attitude toward daycare versus newer city parents’ higher expectations and demands
		Newer city parents’ sensitivities and struggles to adjust to a new area stabilize
Cognitive changes	Pursuing intrinsic motivation	Pursue intrinsic motivation rather than extrinsic motivation, such as what others think of you
	Change in value	Prioritize inner happiness over material success
Emotional changes	The courage of self-acknowledgment	The courage to move beyond the paralyzing feelings of self-doubt and accept yourself as you are
Behavioral changes	Pursuing work–life balance	Balancing work and life
Impostor phenomenon persists	Maintaining impostor phenomenon	Continue to hide your shortcomings as your responsibilities increase
		Constant awareness and nervousness about negative parental evaluations
	Reduced impostor phenomenon	Finding psychological relief from the judgment of others
		Recognize your value

## Data Availability

The data presented in this study are available on request from the corresponding author due to privacy and ethical reasons.
